# Strategies to reduce safety violations for working from heights in construction companies: study protocol for a randomized controlled trial

**DOI:** 10.1186/1471-2458-14-541

**Published:** 2014-05-31

**Authors:** Henk F van der Molen, Monique HW Frings-Dresen

**Affiliations:** 1 Coronel Institute of Occupational Health, Academic Medical Center, University of Amsterdam, P.O. Box 22660, Amsterdam 1100 DD, the Netherlands; 2 Arbouw, P.O. Box 213, Harderwijk 3840 AE, the Netherlands

**Keywords:** Safety hazards, Working from heights, Safety behavior, Construction industry

## Abstract

**Background:**

Safety measures should be applied to reduce work-related fatal and non-fatal fall injuries. However, according to the labor inspectorate, more than 80% of Dutch construction sites violate safety regulations for working from heights. To increase compliance with safety regulations, employers and workers have to select, implement and monitor safety measures. To facilitate this behavioral change, stimulating knowledge awareness and personalized feedback are frequently advocated behavior change techniques. For this study, two behavior change strategies have been developed in addition to the announcement of safety inspections by the labor inspectorate. These strategies consist of 1) face-to-face contacts with safety consultants and 2) direct mail with access to internet facilities. The objective of this study is to evaluate the effectiveness of these two strategies on the safety violations for working from heights, the process and the cost measures.

**Methods/Design:**

This study is a block randomized intervention trial in 27 cities to establish the effects of the face-to-face guidance strategy (N = 9), a direct mailing strategy (N = 9) and a control condition of no guidance (N = 9) on safety violations to record by labor inspectors after three months. A process evaluation for both strategies will be performed to determine program implementation (reach, dose delivered and dose received), satisfaction, knowledge and perceived safety behavior. A cost analysis will be performed to establish the financial costs for both strategies. The present study is in accordance with the CONSORT statement.

**Discussion:**

This study increases insight into performing practice-based randomized controlled trials. The outcome will help to evaluate the effect of two guidance strategies on safety violations. If these strategies are effective, implementation of these strategies through the national institute of safety and health or labor inspectorate can take place to guide construction companies in complying with safety regulations.

**Trial registration:**

NTR 4298 on 29-nov-2013.

## Background

Worldwide, occupational diseases and injuries frequently occur and are consequences of exposures exceeding biological tolerances of humans. The burden of occupational diseases and injuries is high, especially in the construction industry [1,2]. Construction workers are frequently exposed to various types of injury-inducing hazards, especially falling from heights [3]. In principle, occupational diseases [4-6] and occupational injuries can be prevented by means of control measures at worksites.

In the construction industry – as in many other industrial sectors – the majority of control measures are not evaluated in terms of reductions in injuries [7,8]. The reality in daily practice is that work-related injuries occur too infrequently to set up (randomized) controlled intervention studies with injuries as primary outcome measure. In addition, it is difficult and costly to effectively intervene across a large enough construction population. The present randomized controlled intervention study focuses on a more frequently occurring outcome measure like safety violations in the construction industry, accompanied by process measures to get insight in the implementation to evaluate the effect of two strategies.

According to the labor inspectorate (personal communication), more than 80% of Dutch construction sites violate safety regulations for working from heights, while 14% of the construction workers report that unsafe situations prevail at work [9]. Safety measures should be applied to reduce work-related fatal and non-fatal fall injuries. To increase compliance with safety regulations, employers and workers need to select, implement and monitor safety measures. To facilitate this behavioral change [10], stimulating knowledge awareness (e.g. Leeman et al. [11]) and personalized feedback (e.g. Diclemente et al. [12]) are behavior change techniques that are frequently advocated.

For this study, two behavior change strategies have been developed to reduce the number of safety violations concerning the installation and use of rolling scaffolds, ladders and stairs, in addition to the announcement of safety inspections by the labor inspectorate. These strategies are based on aspects of awareness-raising and personalized feedback and consist of 1) face-to-face contacts with safety consultants and 2) direct mail with internet links.

### Study objectives

The objective of this study is to evaluate the effectiveness of a face-to-face strategy and a direct mail strategy on safety violations for working from heights among construction companies compared to the control condition of only the announcement of safety inspections by the labor inspectorate. For both guidance strategies, a process and cost evaluation will be performed.

### Hypothesis

We hypothesize that both guidance strategies will reduce safety violations with rolling scaffolds, ladders and stairs compared to the control condition of a general announcement of inspection in construction companies. In addition, the face-to-face strategy is thought to be superior to the direct mail strategy. This is expected to be due to a higher impact of personalized feedback in the face-to-face guidance strategy on executing safety measures within the construction companies compared with awareness-raising in the direct mail strategy. The financial costs are expected to be higher in the face-to-face guidance strategy, especially due to hiring safety consultants and their coordinator for work site visits.

## Methods/Design

In a three-armed block randomized controlled trial (RCT), 27 large cities has been stratified within three regions in the Netherlands (North-East, West, South) and each city has been assigned to one of two intervention groups or the control group using nQuery Advisor® Version 7.0. As a result of the inspection procedure of the Dutch labor inspectorate, i.e. unannounced work site inspections in a well-defined area and time period in the Netherlands, the interventions take place at construction companies working in larger cities.

For the description of the design of the safety intervention and the two guidance strategies, we follow the CONSORT statement [13].

### Study design

A randomized intervention trial is performed to compare the effectiveness of two behavior change strategies, a face-to-face guidance strategy and a direct mailing strategy, with a control condition. A process evaluation and a cost evaluation will take place for both guidance strategies.

The study protocol does not meet the criteria of the “Act medical-scientific research with human participants”, i.e. not a study of a medical nature and the subjects receive not a particular treatment or are asked to behave in a particular way [14]. Therefore, the Medical Ethics Committee of the Academic Medical Center Amsterdam (AMC) has not been asked for approval of the study protocol.

### Setting

The guidance strategies will be given to construction companies. Inclusion criteria of the construction companies are: 1) involved in the painting and maintenance of buildings and 2) working in one of the 18 pre-randomized bigger cities in the Netherlands during May 2014.

### Study population

The research population includes all construction sites of the participating companies.

### Recruitment of participants

Two methods for recruiting the construction companies will be used. First of all, a call centre will contact potential eligible companies located in an area within 50 kilometers of one of the 18 pre-randomized cities and companies with at least 15 workers who are expected to work in a wider area in the Netherlands. The second method of recruitment consists of publishing a call-up with a description of the project in the employers’ newsletter and the website and newsletter of the national organization for safety and health (Arbouw). Construction companies willing to participate are contacted by Arbouw for further arrangements concerning the proposed interventions. Employers and workers will formally be asked for their consent when sending the questionnaires.

### Interventions

The safety measures within the two guidance strategies are based on the inspection module of the labor inspectorate and guidelines of the Dutch construction safety and health institute Arbouw.

#### Face-to-face guidance strategy

The face-to-face guidance strategy consists of personal advice at construction companies with a maximum of three visits in which workers are informed about preventing falling hazards with rolling scaffolds, ladders and stairs. The employer or contact person of each construction company is encouraged to involve all their workers in the selection, use and monitoring of safety measures to reduce falling hazards when working with rolling scaffolds, ladders and stairs.

Company visits will take place at the company or at work sites on dates and times agreed on with the contact person of each company during a three-month period. The number of company visits will depend on the size of the company; one visit when <5 workers, two visits when 5–15 workers, and three visits when > 15 workers. It is advisable to contact management and the workers’ council when planning company visits.

Each visit consists of a one- to two-hour interactive consultancy meeting with the contact person and workers of the construction company. Information is exchanged concerning selection, implementation and monitoring of safety measures with regard to rolling scaffolds, ladders and stairs. The safety consultant writes a short report of the findings and the advised safety measures. A maximum of five types of advice can be given, and advice can be added in subsequent visits to the same company. In a company report, advice is grouped into i) the choice and suitability of the equipment for working from heights (i.e. rolling scaffolds, ladders and stairs), ii) safety measures for risk control when using equipment for working from heights, iii) teaching and training, iv) supervising on the selection and safety use of equipment for working from heights, v) remaining advice for improving safe and healthy workplaces.

In total, six safety consultants, experienced in equipment for working from heights, are involved in the face-to-face strategy. In a meeting, these consultants are informed about documented safety measures in the inspection module of the labor inspectorate, safety protocols of employers’ organization providing equipment for working from heights, and two guidelines of Arbouw concerning safe use of rolling scaffolds, and ladders and stairs.

#### Direct mail guidance strategy

The direct mail guidance strategy consists of sending direct mail to the construction companies and workers in a paint pot where workers are informed about prevention of falling hazards with rolling scaffolds, ladders and stairs. The paint pot contains a poster with URLs for the Internet approach (http://www.schilderenophoogte.nl) to four types of information and instruction materials: brochures and poster, checklists (instructions for safe installation and use of equipment), video and toolbox (to inform and instruct workers during toolbox meeting). A falling paint pot is illustrated on the poster and used as a metaphor for falling hazards. The employer or contact person of each construction company is stimulated to involve all their workers in the selection, use and monitoring of safety measures to reduce falling hazards when working with rolling scaffolds, ladders and stairs.

### Control condition

In the control condition, no guidance strategies will take place, with the exception of a general announcement of inspection in construction companies.

### Outcome measure

Safety violations are the primary outcome measure and are defined as safety violations during the installation and use of equipment for working from heights. The labor inspectorate will check safety violations concerning equipment of rolling scaffolds, ladders and stairs on worksites of painters during a three-week period. The safety violations consist of 0 to 30 safety hazards (see below list). In addition, workers will be asked to specify the reasons for identified violations during the inspections.

### Safety violations

#### General

1. incomplete risk inventory and evaluation

2. no collective safety measures

3. equipment not suitable for type of work

4. no possibility to evacuate

5. extra falling hazard when stepping over

6. defective materials

7. defective construction

8. hazards for falling objects, electricity, heat

9. no annual technical examination

10. bad maintenance

#### Specific for rolling scaffolds

11. not safe to access working floor

12. working floor not completely closed

13. no side safeguarding working floor

14. no safeguarding when climbing floors

15. no (guarantee for) stability or strength testing

16. no (de)installation scheme or authorized

17. unstable on ground surface

18. inadequate angle braces

19. no prescribed stabilizer

20. no prescribed anchoring

21. no safeguarded wheels

22. flooring not adequate or secured

23. no competent worker for installation

24. no instruction for installation

25. climbing outside

26. transporting with persons on it

27. overloading

#### Specific for ladders and stairs

28. unstable during use

29. no horizontal steps

30. no safe grip with hand

### Process evaluation

The process evaluation of the two guidance strategies will be determined using indicators as defined by Linnan and Steckler [15], Murta et al. [16] and Bowen et al. [17]. The following indicators for implementation of the program will be evaluated [15,16]: reach, dose delivered, dose received. Satisfaction with the intervention (score 0–10), increase in perceived knowledge on safety measures (score 0–10) and perceived effectiveness on safety behavior (score 0–10) are used as indicators for acceptability concerning the interventions [17].

Reach is defined as the attendance rate of the construction companies at the intervention. Attendance rate is defined as the number of construction companies participating in this study relative to the number of eligible construction companies invited through the recruitment strategies. The attendance will be assessed by means of a logbook during the recruitment of the construction companies. Construction companies that do not participate will be asked to explain why.

Dose delivered refers to the proportion of the intended intervention that is actually delivered to the participating contact persons of the construction companies. For the face-to-face guidance strategy, the number of company visits and company reports including advice delivered by the safety consultants to the contact person of the construction companies will be assessed by means of a logbook filled in by the safety consultants and checked by Arbouw, who coordinate the company visits as applied by the safety consultants. For the direct mailing strategy, dose delivered will be assessed by means of the number of direct postal mails and emails to the contact person of the construction companies. The dose delivered is rated as sufficient when more than 90% of the companies receive the interventions (i.e. visit of the safety consultant and postal information).

Dose received refers to the proportion of activities in the intervention that are actually performed by the employer and workers of the construction companies. For both intervention strategies, dose received is assessed by three questions to the employer and two questions to the workers in a questionnaire after completion of the intervention period. For the face-to-face strategy, employers are asked if they have received a safety consultant (yes/no), have given feedback of advice to all workers (yes/no) and have taken preventive actions (yes/no; type of actions: education, training, buying safer equipment, supervision, appointment with suppliers, appointment with principal, other). The workers are asked if they have received information (yes/no) and if they have taken preventive actions (yes/no; type of actions: education, training, following equipment instruction, supervision with colleagues, appointment with suppliers, appointment with principal, other). For the direct mailing strategy, the employers are asked if they have read the postal and internet information (yes/no), if they have disseminated information to all workers (yes/no) and if they have undertaken preventive actions (yes/no; type of actions: education, training, buying safer equipment, supervision, appointment with suppliers, appointment with principal, other). The workers are asked if they have received information (yes/no) and if they have taken preventive actions (yes/no; type of actions: informing, training, following equipment instruction, supervision with colleagues, appointment with suppliers, appointment with principal, other).

Satisfaction is measured by asking the employers and workers how they rated their satisfaction with the intervention as a whole and its individual components, on a scale from 0 (not satisfied) to 10 (very satisfied).

Increased knowledge about working safely with rolling scaffolds, ladders and stairs as a result of the intervention is measured by asking the employers and workers to what extent their knowledge increased from (0–10; 0 = not more knowledge, 10 = much more knowledge). The rates are defined as not more knowledge (0), little (≥0 and <6), moderate (≥6 and <7.5) or much more knowledge (≥7.5).

Effect on working safely with rolling scaffolds, ladders and stairs as a result of the intervention is measured by asking the employers and workers to what extent the perceived effect on safety behavior is rated (0–10, 0 = no effect, 10 = large effect). The rates are defined as poor (<6), moderate (≥6 and <7.5) or good (≥7.5).

The process outcomes will be measured after the intervention period through questionnaires sent to the companies and their workers.

### Economic evaluation

A financial cost analysis for each intervention strategy will be made. The costs of the face-to-face strategy will be calculated by multiplying the number of company visits by the fixed cost per visit, plus travel costs, the coordination costs charged by Arbouw and the cost of information materials. The cost of the direct mailing strategy will be calculated by totaling the coordination costs charged by Arbouw, the costs of developing information materials and the website, and the costs of direct mailing.

### Sample size

The sample size is based on detecting an absolute difference in safety violations of 25% between the face-to-face guidance strategy and the control condition, and 15% between the direct mail strategy and the control condition. Data from previous inspection projects on equipment for working from heights revealed that in 84% of the cases safety violations were ascertained. We have decided that a 15-25% absolute reduction in ascertained safety violations concerning equipment for working from heights is a realistic and relevant difference.

Based on a power calculation using the nQuery Advisor® Version 7.0, 64 construction locations involved in painting and maintenance per group must be included to achieve a difference of 15% -25% (from 84% to 69% and 59% respectively) with an alpha of 0.05 (two-tailed) and a power of (1-beta) = 0.80. Based on previous inspections on rolling scaffolds, stairs and ladders, the assumption is made that at least half of the construction sites in the 27 large Dutch cities during May 2014 visited by the labor inspectorate are involved in painting and maintenance. Since the policy of the labor inspectorate is not to make a distinction between painting and non-painting companies, this results in a target for the inspections of 128 work sites per group.

### Randomization

The stratified allocation of the cities (nine cities in each of the three regions) to the intervention groups or to the control group will be performed using a random, computerized allocation procedure. The primary researcher (HM) blindly randomizes the groups for the 27 cities. The safety consultants who execute the face-to-face strategy are assigned by Arbouw.

The intervention and implementation strategy makes blind group assignment of the construction companies, consultants and Arbouw impossible.

### Statistical analyses

The primary outcome measures, i.e. ascertained safety violations, will be presented for the two guidance strategies and the control condition using descriptive statistics. The number of safety violations concerning the installing and use of equipment for working from heights at worksites of painting and maintenance companies ascertained by the Dutch labor inspectorate in the included 27 cities during three weeks in May 2014 will be retrieved by the primary researcher (HM) from the database of the Dutch labor inspectorate. Differences in the mean number of safety violations will be tested using an analysis of variance.

The three process measurements (i.e. satisfaction, insight and safe working behavior) of both intervention strategies are post-tested and converted into continuous variables on a scale of 0–100. The differences between both interventions are examined using independent t-tests.

The analysis is performed on an intention-to-treat basis. IBM SPSS 19.0 will be used for the statistical tests. Statistical significance is defined as p < 0.05 for all outcome measures.

The costs calculation for each intervention strategy will be analyzed descriptively.

## Discussion

The present randomized controlled intervention study focuses on two frequently advocated strategies to reduce safety violations in the construction industry to gain insight into their effect, implementation and acceptability.

The outcome of this study will help to evaluate the effect of two guidance strategies on reducing safety violations when working from heights with rolling scaffolds, ladders and stairs. When these strategies are effective, stimulation of safety measures through the national institute of safety and health or labor inspectorate can take place to encourage construction companies to comply with safety regulations.

### Methodological considerations

For both guidance strategies, the content and approach has been developed by the Dutch institute on safety and health in the construction industry. Therefore, the practice base has been incorporated to the maximum. For instance, in the face-to-face strategy, the safety consultants have experience in safety in construction work; in the direct mailing strategy, the information materials have been adapted to the context of painters. In addition, construction companies are free to choose how they want to control the safety hazards when working with rolling scaffolds, ladders and stairs. This adds to the number of dynamic implementation strategies which could also be evaluated in actual performance, i.e. reduced number of safety violations. Standardizing the function and process of complex interventions [18] in this study, respectively stimulating the implementation of safety measures [function] and awareness-raising [process], and personal feedback [process] enables the evaluation of such interventions through RCTs. This method of standardization allows the incorporation of two important elements in studying implementation strategies in pragmatic trials [16,19]: 1) tailoring intervention components to context-specific needs of construction companies, and 2) determining the effectiveness of both strategies on different process measures.

Four categories of process measures are proposed in implementation research in the construction industry [20]: awareness of risk factors and control measures; attitude toward changing behavior; ability to change behavior, and actually changing and maintaining behavior. In the present study, three out of four of these aspects are measured: increase in knowledge in safe working with rolling scaffolds, ladders and stairs, attitude in terms of satisfaction and perceived effect of the interventions, and behavior in terms of the primary outcome measure of safety violations.

A constraint of this study is that the ability to effect a behavioral change is not explicitly addressed in the interventions and in the process measures, but it is thought that most of the construction workers are able to comply with the safety regulations when working with rolling scaffolds, ladders and stairs. Another constraint is the measurement of safety violations through the inspection procedure of unannounced work site inspections in a well-defined area and time period in the Netherlands. Therefore, a possible lack of power can emerge due to a lack of the intervention companies when the labor inspectorate is inspecting work sites in 18 of the 27 cities. Due to ethical reasons, i.e. introducing unequal risk of incurring financial penalties when safety violations are established by the labor inspectorate, all construction sites in the 27 cities can be the subject of inspection and the researchers will not reveal the names of the 18 intervention cities nor the names of the participating painting companies.

The results of the present study will be available in the second half of 2014.

## Competing interests

The authors declare that they have no competing interests.

## Authors’ contributions

HM conceived and designed the study and drafted the manuscript. MFD participated in the design of the study and the manuscript preparation. Both authors read and approved the final manuscript.

## Pre-publication history

The pre-publication history for this paper can be accessed here:

http://www.biomedcentral.com/1471-2458/14/541/prepub
